# The Power of Negative Affect during the COVID-19 Pandemic: Negative Affect Leverages Need Satisfaction to Foster Work Centrality

**DOI:** 10.3390/ijerph20032379

**Published:** 2023-01-29

**Authors:** Jérémy Toutant, Christian Vandenberghe

**Affiliations:** Department of Management, HEC Montréal, 3000 Côte Ste-Catherine, Montréal, QC H3T 2A7, Canada

**Keywords:** positive and negative affectivity, work centrality, need for autonomy, need for relatedness, need for competence

## Abstract

The COVID-19 pandemic has created unprecedented disruptions in organizations and people’s lives by generating uncertainty, anxiety, and isolation for most employees around the globe. Such disruptive context may have prompted employees to reconsider their identification with their work role, defined as work centrality. As such reconsideration may have deep implications, we reasoned that individuals’ affective dispositions would influence work centrality across time during the pandemic. Drawing upon the broaden-and-build theory of positive emotions and the met expectations underpinnings of negative affectivity, we predicted that positive and negative affect would foster, albeit for different reasons, work centrality. Based on self-determination theory, we further expected the fulfilment of the needs for autonomy, relatedness, and competence to enhance the effect of positive and negative affectivity. Based on a three-wave study (*N* = 379) conducted during the COVID-19 lockdown followed by a reopening of the economy in Canada (i.e., May to July 2020), we found negative affectivity, but not positive affectivity, to drive work centrality over time, and found this effect to be enhanced at high levels of the satisfaction of the needs for autonomy and relatedness. The implications of these results for our understanding of the role of trait affectivity in times of crisis are discussed.

## 1. Introduction

The COVID-19 pandemic outbreak has immersed the world in a health-related and economic crisis whose intensity has not been witnessed for more than a century [1]. For example, before the rise of the pandemic in March 2020, the unemployment rate in the United States was around 3.5% [2], but in early May 2020, over 30 million people lost their jobs [3], representing roughly 19% of the labor force [1]. From a health-related perspective, the COVID-19 pandemic engendered anxiety, uncertainty, loss of control, and feelings of helplessness and distress [4,5]. Most governments around the globe set severe restrictions on their populations, including business shutdowns, to limit the spread of the COVID-19 virus. These restrictions notably involved the prohibition of public gatherings, working from home for those employees eligible for such work conditions, keeping open only the essential businesses, and implementing furloughs with financial support from the governments [6]. In several countries, particularly in Europe and Canada, a period of lockdown of the economy was implemented from mid-March to early May 2020. Other lockdowns occurred later in other countries.

There is evidence that the COVID-19 pandemic has precipitated an average decline in employees’ job attitudes, including affective commitment to their organization [7], possibly due to a variety of factors such as increased stress and work-related loneliness [8], COVID-19-triggered anxiety [9], or lack of support from organizations [10]. However, research suggests that individual differences may exist in how employees react in the context of environmental disruptions. For example, Mihalache and Mihalache [10] found that employees with low levels of core self-evaluation, an aggregate trait reflecting how positively individuals think of themselves and the confidence they have in their abilities [11], experienced more positive change in affective organizational commitment when their organization was perceived to provide support and their supervisor was accessible during the COVID-19 pandemic. Similarly, scholars speculated that extroversion may facilitate adjustment in the face of disruptive events because it is associated with more frequent positive emotions and interpersonal skills [6,12].

The present study extends the research stream on the role of individual differences during the COVID-19 pandemic by examining how positive and negative affectivity relate to work centrality, a construct that reflects the extent to which individuals view their work as a central component of their lives [13,14]. Although work centrality has often been treated as a stable variable [15,16], we adopted a state-like perspective to it as research indicates that disruptive events such as the COVID-19 pandemic may encourage people to reassess the meaning attached to their lives and work [17] and, hence, may modify how central the experience of work is perceived to be. Positive and negative affectivity, as traits that reflect the extent to which people experience positive emotions (e.g., enthusiasm and alertness) and negative emotions (e.g., fear and anger), respectively [18,19], are likely to influence work centrality during the pandemic, yet for different reasons. Specifically, we suggest that positive affectivity may encourage work centrality because it fosters approach behavior (i.e., a tendency to seek rewarding stimuli), and that negative affectivity may do so because it promotes avoidance behavior (i.e., a tendency to avoid aversive stimuli; committing oneself to work would help escape from an environment perceived to be threatening [due to the pandemic]).

Drawing upon self-determination theory (SDT) [20,21], we further aim to explore the role of satisfaction of the basic needs for autonomy, relatedness, and competence, as potential moderators of positive and negative affectivity’s effects on work centrality. The tenets of SDT suggest that the fulfilment of the needs for autonomy (i.e., the need to freely decide one’s own actions), relatedness (i.e., the need to be connected to others), and competence (i.e., the need to experience mastery of one’s tasks) all contribute to increase work motivation and human flourishing [22,23]. We contend that the expected positive effects of positive and negative affectivity on work centrality during the COVID-19 pandemic are potentially influenced by the extent to which the basic psychological needs are fulfilled. In other words, the satisfaction of basic needs at work would represent a key boundary condition associated with the relation between trait affectivity and work centrality. For example, if the COVID-19 pandemic comes to reduce the ability of people to autonomously organize their work and maintain a sense of control over their work schedule, limit their ability to be connected to others, or decrease the opportunities to learn new things, the needs for autonomy, relatedness, and competence, respectively, would be thwarted [8]. In such circumstances, the expected benefits that may be afforded by high levels of positive and negative affectivity in terms of increased work centrality may be reduced due to need frustration. The reverse would be true when the three basic needs are fulfilled.

The present study contributes to the literature in several important ways. First, research on employee outcomes during the COVID-19 pandemic has largely explored how variables related to the work context have influenced employee well-being and reactions. For example, employee attitudes and health outcomes during the pandemic have been examined in connection to perceived job control and work-related loneliness [8], supervisors’ servant leadership behavior [9], workplace support [10], COVID-19 information exposure [24], COVID-19 induced work stressors [4], and COVID-19-triggered health anxiety [5]. However, surprisingly, little emphasis has been placed on scrutinizing whether individual difference variables may explain employee outcomes. The present study counts among the few attempts at exploring how personality, as captured by positive and negative affectivity, impacts employee work centrality. As it stands, the literature has essentially focused on the positive virtues of positive affectivity in terms of job attitudes and performance and has reported negative affectivity to be associated with negative outcomes (e.g., high emotional exhaustion, low job satisfaction) [25,26]. Our study challenges these prior findings by demonstrating that the COVID-19 pandemic provides a specific context that alters the natural functioning of trait affectivity. Second, we introduce a state-like perspective to work centrality, a construct traditionally seen as a dispositional variable. In doing so, we assume that the disruptive nature of the pandemic context has shocked individuals to a point that revisions to their beliefs about the centrality of work in their lives may be affected. This assumption is based on the specific characteristics of the pandemic, as compared to other traumatic events such as wars, natural disasters, or terrorist attacks. Contrary to other traumatic events, the COVID-19 pandemic (a) has caused huge disruptions in healthcare, (b) has affected populations worldwide, (c) has led to amplified stress due to extended media coverage [27], (d) and is a global event requiring years-long responses. Third, we examine the fulfilment of basic needs as boundary conditions associated with the effects of positive and negative affectivity. As such, we break new ground by acknowledging that the work context itself interacts with trait affect to provide opportunities at varying degrees for individuals to satisfy their needs for autonomy, relatedness, and competence.

In the next section, we present and discuss our hypotheses. Next, we present the results of a three-wave, monthly study aimed at examining our hypotheses, which was conducted from May to July 2020 during a period characterized by first a lockdown, followed by the progressive reopening of the economy in Canada.

### 1.1. Positive and Negative Affectivity and Work Centrality

Watson and colleagues have provided consistent evidence for the existence of a two-dimensional structure of affect [19,28]. People with high levels of positive affectivity display a dispositional tendency to experience feelings such as alertness, enthusiasm, and activeness, while people with high levels of negative affectivity are inclined to experience emotions such as guilt, nervousness, fear, and anger [26,28]. High positive affectivity is also associated with the search for comradeship with other people, a positive view of oneself, and positive connections to the environment [26]. In contrast, negative affectivity relates to negative views of the self, pessimistic evaluations of the environment, and sensitivity to work stress [29,30]. Positive and negative affectivity are also related to the operation of two distinct neurobiological systems that regulate approach and avoidance behavior, respectively [31]. Approach motivation, as illustrated by positive affectivity, is generated by the behavioral activation system, while avoidance motivation, as embodied by negative affectivity, is managed by the behavioral inhibition system [32,33]. This may explain why some people are drawn to rewards, opportunities, and incentives (i.e., behavioral activation) while others are sensitized to threats from the environment and exhibit an escape action tendency (i.e., behavioral inhibition) [18,31].

Although the above discussion indicates that positive and negative affectivity are associated with different perceptions of the world and action tendencies, we argue that both constructs should contribute to enhance work centrality during the COVID-19 pandemic, but through different mechanisms. First, the broaden-and-build theory, e.g., [18,34], suggests that positive affectivity exerts effects on favorable job attitudes through an approach motivation because it builds individuals’ resilience, e.g., [18,34,35]. Specifically, people with high levels of positive affectivity tend to perceive opportunities in their environment and are biased toward to encoding and recovering positive events and information about their life [30]. These individuals broaden their attention and develop positive relationships with others [36], and as they tend to experience more frequent positive emotions they are able to build and replenish their regulatory resources, e.g., [37], which make them more optimistic and resilient in the face of disruptive events [38]. This is because positive affectivity helps people engage in exploration of the environment and build their competencies and self-efficacy [39]. Research has, indeed, reported that positive emotions result in a broadened scope of attention [40], greater mindfulness [37], and greater self-efficacy [39].

In the context of the pandemic, where stressful work conditions emerge due to anxiety triggered by the COVID-19 [5], it is likely that people with high levels of positive affectivity can better cope with these adverse conditions. They may, particularly, maintain their approach motivation by focusing on their work and keeping it as a central activity in their life, even though they are exposed to anxious information regarding the spread of the virus and receive regular updates about the death toll [24]. Indeed, COVID-19 information exposure may increase anxiety [9,24], hence, distracting the attention of people regarding the importance of work in their life, and plausibly reduce the extent to which they identify with their work role, i.e., a key feature of work centrality [15]. One could thus expect that the anxiety generated by the COVID-19 pandemic would reduce the resources people could normally devote to their work [13]. However, following the tenets of the broaden-and-build theory of positive emotions, e.g., [38], people with high levels of positive affectivity may enjoy more regulatory resources to face the pandemic. Their resources may feed an approach motivation toward work which helps maintain work as a central activity. In other words, these people should be able to maintain an approach motivation toward work owing to their resilience, optimism, and resources, despite the adverse conditions created by the pandemic. Therefore, the following hypothesis is proposed.

**Hypothesis** **1a.** *Positive affectivity is positively related to work centrality during the COVID-19 pandemic.*

The tenets of the broaden-and-build theory also suggest that negative affectivity, which includes the experience of frequent negative emotions such as anger and fear [26], narrows people’s scope of attention by leading them to focus on specific threats identified in the environment. In Fredrickson’s [18] terms, negative emotions instill flight-or-fight behaviors “by calling to mind an urge to act in a particular way (e.g., escape, attack, expel)” when confronted with threatening situations. It has been established for a long time that negative affectivity is associated with work-related stress, e.g., [41], and that avoidance motivation is a central motive underlying this trait [31]. This may explain why people with high negative affectivity tend to view the world in negative terms and develop negative expectations regarding the occurrence of threats in the environment [30,42].

However, despite the negative outcomes generally associated with negative affectivity [25,26], the COVID-19 pandemic may offer opportunities for people to take benefits from this trait. Specifically, we identify two reasons why the disruptive context of the pandemic may turn negative affectivity into an asset when it comes to predicting individuals’ identification with their work role. First, people high in negative affectivity hold negative expectations about how the world evolves and generally expect negative things to occur [43,44]. Thus, the outbreak of the COVID-19 pandemic may have acted as a confirmation of their negative expectations, making them less surprised and concerned by the COVID-19 disease compared to people with low levels of negative affectivity. We suggest that, for people with high negative affectivity, the emergence of the pandemic, as a negative event, acts as a confirmation of the negative expectations they entertain about their environment. They may then feel that their vigilance about the occurrence of threatening events (i.e., the pandemic) is justified. For example, Begley and Lee [43] found that employees with high negative affectivity were less disappointed by the receipt of small bonuses (i.e., a negative event) than people low in negative affectivity. Thus, following a met expectations mechanism [45], individuals high in negative affectivity may take advantage of their pessimistic view of the world and feel comfortable spending time working, and invest resources in their work role. Second, this leads us to consider another mechanism by which negative affectivity may foster work centrality: by focusing on their work during the COVID-19 pandemic, people high in negative affectivity may escape from a threatening environment, thereby illustrating the *flight* or avoidant behavior typically associated with this trait [18,38]. The above discussion leads to the following hypothesis.

**Hypothesis** **1b.** *Negative affectivity is positively related to work centrality during the COVID-19 pandemic.*

### 1.2. The Moderating Role of Basic Needs Satisfaction

We suggest that the association between positive and negative affectivity and work centrality should be contingent on the satisfaction of basic psychological needs (i.e., autonomy, relatedness, and competence). Following the tenets of SDT [46], the satisfaction of the need for autonomy allows individuals to experience freedom when completing their work duties and the sense that they are owners of their work, which is fundamentally gratifying [21,47] and fosters growth and well-being [22]. Thus, satisfying the need for autonomy results in experiencing a sense of choice and volition when completing one’s tasks [46]. The need for relatedness refers to the need to be connected to others, and to be loved and care for others [22,48]. This need is fulfilled when people develop meaningful and close relations to others and its satisfaction leads to optimal psychological functioning and growth [21]. Finally, the need for competence refers to the need to develop a sense of mastery over one’s tasks, explore the environment, and expand one’s skills. The fulfilment of the need for competence enhances intrinsic motivation and self-efficacy [21].

The fulfilment of each of the three basic needs may amplify the expected positive effects of both positive affectivity and negative affectivity on work centrality during the COVID-19 pandemic. Research has, indeed, shown that basic needs satisfaction enhances psychological growth, well-being, satisfaction, and internalization [20,22,49]. For example, individuals with high levels of positive affectivity may experience greater resilience from seeing their basic needs satisfied at work. We thus argue that need satisfaction may expand the psychological growth of these individuals, which would increase the salience of work as a central activity in their life. This expectation is in line with the broad-and-build theory [18], which basically states that any environmental factor that increases people’s resilience and resources would contribute to enhance the benefits of positive emotions. Similarly, need fulfilment should represent an important boundary condition for negative affectivity. People with high negative affectivity expect negative things to happen in their environment [43,50], of which the COVID-19 pandemic yields confirmation. This may push them to think that investing resources into their work would be the best option in the context of the pandemic. If, at the same time, their work fulfills their basic needs for autonomy, relatedness, and competence, their choice of focusing on their work role would be further appreciated and confirmed. Overall, the above discussion leads to the following hypotheses.

**Hypothesis** **2.** *The satisfaction of the needs for autonomy (Hypothesis 2a), relatedness (Hypothesis 2b), and competence (Hypothesis 2c) will moderate the relationship between positive affectivity and work centrality during the COVID-19 pandemic, such that this relationship is stronger (more positive) when the basic needs are satisfied.*

**Hypothesis** **3.** *The satisfaction of the needs for autonomy (Hypothesis 3a), relatedness (Hypothesis 3b), and competence (Hypothesis 3c) will moderate the relationship between negative affectivity and work centrality during the COVID-19 pandemic, such that this relationship is stronger (more positive) when the basic needs are satisfied.*

Our hypotheses were tested through a three-wave, monthly study, partly conducted during the COVID-19 lockdown in Canada, from early May 2020 to early July 2020. Participants reported their level of positive and negative affectivity at Time 1, work centrality at Time 2 (as a control variable), and satisfaction of their needs for autonomy, relatedness, and competence, and work centrality, at Time 3.

## 2. Materials and Methods

### 2.1. Participants and Procedure

As part of a larger longitudinal project, we recruited participants through Delvinia’s (http://www.delvinia.com accessed on 1 November 2020) crowdsourcing survey platform. Delvinia is a Canadian research and data collection organization that owns an online panel with several tens of thousands of panelists representative of the Canadian population in terms of age, gender, education level, and income. Research has established that online platforms provide data as reliable and valid as traditional survey methods, e.g., [51]. An accompanying letter described the research objectives, assured participants that their responses would be confidentially treated, and specified that the research project was longitudinal, with monthly surveys to be completed over a period of six months. In this article, three waves of data collection were targeted (i.e., 1 May, 1 June, and 1 July 2020). In total, 882 participants completed the Time 1 survey while, among them, 619 filled out the Time 2 survey and 379 responded at Time 3 (for an overall response rate of 43%). Thus, the final sample for the study included 379 matched participants across time. Note that, per Delvinia’s survey procedure, participants who failed the attention check item at each measurement time were dropped [52].

Among the Time 1 respondents, average age was 48.10 years (*SD* = 11.25), 49.40% were female, and 64.10% had full-time employment. Education level was distributed as follows: high school (13.80%), college (30.70%), undergraduate (26.10%), certificate (10.90%), master’s (15.00%), doctorate in medicine, dentistry, veterinary, or optometry (1.20%), and Ph.D. (2.30%). Moreover, 89.9% of the respondents were Caucasian, 34.2% were unmarried, 34.8% had one or more child(ren), and 63.4% had a household income between CAD 50,000 and CAD 150,000. Participants were working in various industries including professional, scientific, and technical services (12.0%), educational services (9.6%), health care and social assistance (8.6%), utilities (6.8%), finance and insurance (6.1%), and public administration (5.1%).

We used logistic regression to examine whether respondent attrition across time was randomly distributed [53]. Specifically, the logistic regression model predicted the probability of the respondents staying in (=0) vs. dropping from (=1) the sample at Time 2 or Time 3. The regression model predicting the likelihood of leaving the sample at Time 2 from Time 1 predictors (i.e., age, gender, education level, employment status, positive affectivity, and negative affectivity) was significant, *χ*^2^(6) = 17.27, *p* < 0.01. Age (*B* = −0.02, *p* < 0.05) reduced, while positive affectivity (*B* = 0.28, *p* < 0.01) increased the likelihood of leaving the sample at Time 2. Similarly, the regression model predicting the likelihood of leaving the sample at Time 3 from Time 1 (i.e., age, gender, education level, employment status, positive affectivity, and negative affectivity) and Time 2 (i.e., work centrality) predictors was significant, *χ*^2^(7) = 27.44, *p* < 0.001. Age (*B* = −0.02, *p* < 0.05) reduced while being female (*B* = 0.46, *p* < 0.01) and positive affectivity (*B* = 0.33, *p* < 0.001) increased the likelihood of leaving the sample at Time 3. As age and gender were used as controls, their effects on attrition across time may not be concerning. However, as positive affectivity was a substantive variable, hypothesis testing related to it may have been affected due to range restriction. We discuss these attrition biases in the limitations section.

### 2.2. Measures

#### 2.2.1. Positive and Negative Affectivity

We measured positive and negative affectivity at Time 1 using Thompson’s [54] 5-item, short versions of Watson et al.’s [28] original 10-item scales. Sample items are “In general, I feel determined” (positive affectivity; α = 0.86) and “In general, I feel upset” (negative affectivity; α = 0.82). Items were rated on a scale ranging from 1 (never) to 7 (all the time).

#### 2.2.2. Basic Need Satisfaction

We assessed the satisfaction of the three basic needs for autonomy, relatedness, and competence at Time 3 using the 4-item scale versions of Chiniara and Bentein [55], which were shortened from the corresponding 6-item scales of work-related basic need satisfaction developed by Van den Broeck et al. [47]. A sample item for the autonomy need satisfaction scale is “The degree of freedom I have to do my job the way I think it can be done best” (4 items; α = 0.94), a typical item for the relatedness need satisfaction scale is “The positive social interactions I have at work with other people” (4 items; α = 0.92), while an example item for the competence need satisfaction scale is “The level of mastery I can achieve at my task” (4 items; α = 0.96). Respondents were instructed to refer to the last four weeks while rating the items on a scale ranging from 1 (very dissatisfied) to 7 (very satisfied).

#### 2.2.3. Work Centrality

We measured work centrality at Time 3 using a 3-item scale from Bal and Kooij [56], which was a shortened version of Hirschfeld and Feild’s [14] scale. Two items were positively worded (i.e., “The major satisfaction in my life comes from my job”; “The most important things that happen to me involve my work”), while the third item (i.e., “I have other activities more important than my work”) was reverse coded. As the latter item significantly reduced the reliability of the scale, we dropped it and retained the two positively worded items. Respondents were invited to rate the items by referring to how they felt on the job during the last four weeks, using a scale ranging from 1 (never) to 7 (all the time). The alpha coefficient for the 2-item scale was 0.92.

#### 2.2.4. Control Variables

We controlled for participants’ age, gender, education level, and employment status as, arguably, these variables might be related to work centrality, e.g., [56,57]. Moreover, to make hypothesis testing more robust, we controlled for Time 2 work centrality, which resulted in our tests of hypotheses involving predicting change in work centrality between Time 2 and Time 3. Time 2 work centrality was measured using the same two items as for Time 3 work centrality. The alpha coefficient for Time 2 work centrality was 0.93.

## 3. Results

### 3.1. Confirmatory Factor Analyses

We first examined the dimensionality of our theoretical model using confirmatory factor analysis (CFA) through LISREL 8.80 [58], with the maximum likelihood method of estimation and a covariance matrix as input for the analyses. The following fit indices were used to examine model fit: the non-normed fit index (NNFI), the comparative fit index (CFI), the root mean square error of approximation (RMSEA), and the standardized root mean square residual (SRMR). For the NNFI and CFI values over 0.90 are considered as reflecting good fit, while for the RMSEA and SRMR values below 0.08 indicate good fit [59]. Nested CFA models were compared using chi-square difference tests [60]. The results are presented in Table 1.

The theorized seven-factor model comprising positive and negative affectivity, Time 2 work centrality, satisfaction of the three basic needs, and Time 3 work centrality, yielded a good fit to the data: *χ*^2^(278) = 734.67, *p* < 0.01, NNFI = 0.96, CFI = 0.97, RMSEA = 0.065, SRMR = 0.044. This model also outperformed more parsimonious models that merged two or more factors, such as a series of six-factor models that merged either (a) positive and negative affectivity, Δ*χ*^2^(6) = 562.63, *p* < 0.001, (b) Time 2 and Time 3 work centrality, Δ*χ*^2^(6) = 319.86, *p* < 0.001, (c) Time 3 autonomy need satisfaction and Time 3 work centrality, Δ*χ*^2^(6) = 675.54, *p* < 0.001, (d) Time 3 relatedness need satisfaction and Time 3 work centrality, Δ*χ*^2^(6) = 667.90, *p* < 0.001, or (e) Time 3 competence need satisfaction and Time 3 work centrality, Δ*χ*^2^(6) = 702.70, *p* < 0.001, and (f) a five-factor model where the satisfaction of all three basic needs represented one factor, Δ*χ*^2^(11) = 1389.36, *p* < 0.001. Note that the one-factor CFA did not converge (see Table 1). The seven-factor CFA model is, thus, retained as the best model for subsequent analyses. The completely standardized factor loadings for the scale items associated with the seven-factor CFA model are reported in Table 2.

### 3.2. Descriptive Statistics and Correlations

The descriptive statistics and correlations for the study variables are reported in Table 3. While Time 1 positive affectivity was unrelated to Time 3 work centrality (*r* = 0.02, *ns*), Time 1 negative affectivity was positively related to it (*r* = 0.19, *p* < 0.01). Moreover, Time 3 satisfaction of the needs for autonomy (*r* = 0.25, *p* < 0.01), relatedness (*r* = 0.26, *p* < 0.01), and competence (*r* = 0.17, *p* < 0.01) were all positively related to Time 3 work centrality.

### 3.3. Hypothesis Testing

Our hypotheses related to the prediction of Time 3 work centrality were tested using moderated multiple regression. Control variables (age, gender, education level, employment status, and Time 2 work centrality) were entered in the first step. Then, positive affectivity and negative affectivity, centered to the mean [61], were entered in the second step. We then alternatively entered the satisfaction of each of the three basic needs, which were also centered, in step 3. Finally, the interactions between positive affectivity and negative affectivity and the relevant basic need satisfaction were entered in step 4. Results are reported in Table 4.

Hypotheses 1a,b predicted a positive relationship between Time 1 positive affectivity and Time 1 negative affectivity, respectively, and Time 3 work centrality. As can be seen from Table 4, controlling for Time 2 work centrality, Time 1 positive affectivity was unrelated (*β* = −0.01, *ns*), while Time 1 negative affectivity was positively related (*β* = 0.10, *p* < 0.05) to Time 3 work centrality (Table 4, Step 2). Hypothesis 1a is, thus, rejected, while Hypothesis 1b is supported. Hypotheses 2a-c stated that the satisfaction of the needs for autonomy (Hypothesis 2a), relatedness (Hypothesis 2b), and competence (Hypothesis 2c) would moderate the relationship between Time 1 positive affectivity and Time 3 work centrality such that this relationship would be stronger at higher levels of the need’s satisfaction. Table 4 (Step 4) indicates that, controlling for Time 2 work centrality, none of the interactions between positive affectivity and needs satisfaction were significant (need for autonomy: *β* = 0.02, *ns*; need for relatedness: *β* = 0.02, *ns*; need for competence: *β* = 0.04, *ns*). Therefore, Hypotheses 2a–c are rejected.

Hypotheses 3a–c predicted that the satisfaction of the needs for autonomy (Hypothesis 3a), relatedness (Hypothesis 3b), and competence (Hypothesis 3c) would moderate the relationship between Time 1 negative affectivity and Time 3 work centrality such that this relationship would be stronger at higher levels of the need’s satisfaction. Table 4 (Step 4) shows that controlling for Time 2 work centrality, Time 3 autonomy need satisfaction and relatedness need satisfaction interacted significantly with Time 1 negative affectivity in predicting Time 3 work centrality (*β* = 0.10, *p* < 0.05, and *β* = 0.13, *p* < 0.01, respectively). In contrast, the interaction involving Time 3 competence need satisfaction was non-significant (*β* = 0.07, *ns*) (Table 4, Step 4). Thus, Hypothesis 3c is rejected.

**Figure 1:**
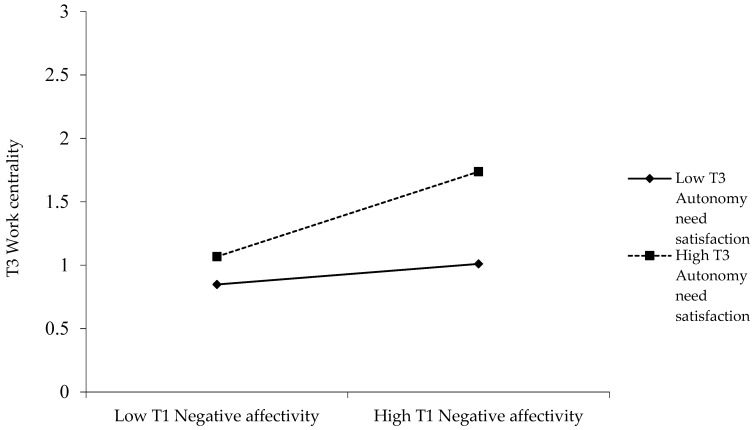
Interaction between Time 1 Negative Affectivity and Time 3 Autonomy Need Satisfaction predicting Time 3 Work Centrality.

**Figure 2:**
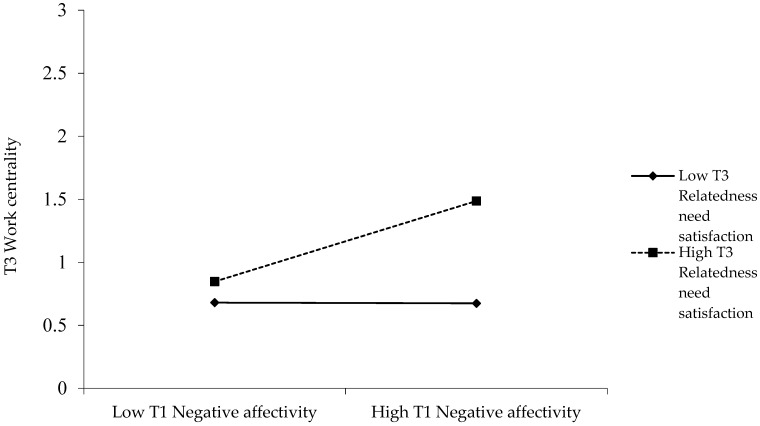
Interaction between Time 1 Negative Affectivity and Time 3 Relatedness Need Satisfaction predicting Time 3 Work Centrality.

To illustrate the pattern of the interactions between negative affectivity and autonomy and relatedness need satisfactions, we conducted simple slopes tests for the relationship between Time 1 negative affectivity and Time 3 work centrality, at one standard deviation above vs. below the mean of the need’s satisfaction [61]. Figure 1 and Figure 2 illustrate these effects. Time 1 negative affectivity was positively related to Time 3 work centrality at high levels of autonomy (*t* [377] = 3.95, *p* < 0.001) and relatedness (*t* [377] = 4.73, *p* < 0.001) need satisfaction, but was non-significant at low levels of the satisfaction of these needs (*t* [377] = 0.96, *ns*, and *t* [377] = −0.04, *ns*, respectively). Moreover, the difference between the slopes was significant in both cases (*t* [377] = 2.17, *p* < 0.05, and *t* [377] = 3.86, *p* < 0.001, respectively). Therefore, Hypotheses 3a and 3b are supported.

## 4. Discussion

Based on the theoretical underpinnings associated with positive and negative affectivity, we hypothesized that each of these traits would provide the needed psychological resources for expecting them to drive work centrality during the disruptive times of the COVID-19 pandemic. We further posited that the fulfillment of employees’ basic needs for autonomy, relatedness, and competence would strengthen the relationship between positive and negative affectivity and work centrality. A multi-wave, monthly study partly conducted during the COVID-19 lockdown in Canada indicated that negative affectivity, not positive affectivity, was a significant and positive predictor of work centrality. This relationship was further strengthened when the basic needs for autonomy and relatedness, but not competence, were fulfilled. These findings highlight the unforeseen benefits of negative affect and the added value of the satisfaction of the needs for autonomy and relatedness in times of crisis. The implications of these results are outlined and discussed in the next sections.

### 4.1. Positive and Negative Affectivity and Work Centrality during the COVID-19 Pandemic

Controlling for the baseline levels of work centrality, negative affectivity was found to positively predict subsequent changes in work centrality. As we suggested, the particular properties of negative affectivity, which include a negative perception of the environment and an anxious anticipation of potential threats to survival [18,50], may constitute psychological resources that help cope with disruptive events. Indeed, the negative perceptual bias and expectations of these individuals [30,43] may provide a sense of confirmation that their vigilance and alertness regarding the occurrence of negative events such as the pandemic outbreak were justified. This may have increased their confidence in their own judgment about the world. As negative affectivity enhanced work centrality over time, it is likely that people with high negative affectivity experienced their work as a haven where they could invest resources. Another interpretation is that, because these individuals are drawn to escape situations perceived to be threatening [19,31], committing to work during the COVID-19 pandemic was a flight behavior whose purpose was to escape from an anxiety-inducing environment. Both interpretations may explain why negative affectivity drove work centrality during the pandemic. However, given the specific features of the COVID-19 pandemic, namely the fact that it has negatively affected populations’ health worldwide and that media coverage around the globe has amplified COVID-19-related anxiety [9], we do not expect that negative affectivity would exert similar effects in a “no-crisis” situation.

Surprisingly, we did not find support for the hypothesis that positive affectivity leads to more work centrality during the COVID-19 pandemic. This is at odds with the tenets of the dominant framework that has been developed to describe the virtuous properties of positive affect, i.e., the broaden-and-build theory [18,35,38]. This theory suggests that positive emotions broaden people’s scope of attention, help them gain support from others, and build their resilience over time [62]. Unexpectedly, these virtuous properties did not suffice to bring benefits in terms of increased work centrality in the context of the COVID-19 pandemic. One may speculate that, despite these positive features, positive affectivity may have downsides in this particular context. For example, the typical optimism of individuals with high positive affectivity may have suffered from the continuous exposure to information regarding the significant increase of infections and deaths, particularly during the first few months after the COVID-19 pandemic outbreak [9]. Therefore, whatever people’s level of positive affectivity, the increased exposure to information about the pandemic may have increased everyone’s death awareness, making it a proximal experience [24]. In such conditions, the underlying optimism associated with positive emotions may have been inefficient. It is even likely that the natural optimism of people with high positive affectivity may have been disconnected with the reality during the pandemic. We know that positive emotions tend to help people experience personal growth and accumulate resources, even in adverse situations [18,35]. However, it is likely that such benefits may not be maintained once people face extreme situations where negative events, such as death exposure and threatening information, are ubiquitous [9]. Another potential explanation is that individuals with high levels of positive affectivity may have focused on other life domains than work per se. As these individuals are efficient at developing relationships with others and building resources from their network [19,63,64], their attention was potentially drawn to their close friends and family rather than to the work role. Re-orienting their attention to other life domains may have helped them maintain their optimistic view of the world. These competing forces may have rendered positive affectivity’s influence on work centrality more diffuse.

### 4.2. Basic Needs Satisfaction during the COVID-19 Pandemic

Although the satisfaction of all three basic needs was positively associated with (Time 3) work centrality, the fulfilment of only two of these needs moderated the relationship between negative affectivity and change in work centrality. Specifically, the fulfilment of the need for autonomy and relatedness, but not competence, enhanced the positive relationship between negative affectivity and work centrality. These findings are consistent with SDT [20], as they illustrate that work environments that provide opportunities for individuals to fulfill their needs for ownership of their jobs and relationships with others foster the likelihood that they identify with their work role when they exhibit high levels of negative affect. In contrast, competence need satisfaction did not moderate the effect of negative affectivity. In retrospect, this may be explained by the fact that the need for competence is less dependent on the environment and partly corresponds to an inherent disposition of individuals to learn new things [22]. Moreover, the needs for autonomy and relatedness have been found to be malleable and the need for competence to be more stable over time [65]. Interestingly, in their meta-analytic review, Van den Broeck et al. [22] found that the satisfaction of the need for competence did not predict work engagement beyond the satisfaction of the needs for autonomy and relatedness. This could explicate why the fulfilment of this need did not make a difference to the relationship between negative affectivity and work centrality.

Although a recent macro theory of positive functioning has been proposed that integrates the tenets of SDT with those of the broaden-and-build theory [66], where positive affect is thought to be a central outcome, the present findings indicate that the satisfaction of the needs for autonomy and relatedness act as boundary conditions for negative affectivity’s relation to work centrality. This suggests that positive affect is plausibly not the key mechanism at play. Rather, as discussed above, vigilance and alertness regarding what is going on in the environment and anticipation of negative things about the COVID-19 infections may have prepared people with high negative affectivity to safely (re-)focus on their work role and see more benefits in developing a sense of ownership and connection to others through their work. However, such a process may not involve positive affect, which is at odds with findings from studies conducted within the perspective of the broaden-and-build theory, e.g., [67].

### 4.3. Limitations and Future Directions

As with all studies, the present research has limitations. First, although we controlled for several factors that may have acted as confounders (i.e., age, gender, education level, and employment status; only education level had a significant and positive effect on change in work centrality), our study was conducted within a specific temporal context. Specifically, our study started (i.e., Time 1: early May 2020) when the country was still in the period of COVID-19 lockdown, and participants were nearly all furloughed at that time. At the subsequent times (Time 2: early June; Time 3: early July), the economy had been nearly fully reopened and participants went back to their jobs. Therefore, the notion of work centrality as it was measured at Time 3 was, plausibly, strengthened by the mere fact that people could return to their jobs, as the lockdown was over. Second, we theorized that people with high levels of negative affectivity plausibly drew negative expectations about the spread of the COVID-19 virus and the related death toll over time, and ultimately experienced a confirmation of these expectations when the lockdown was implemented. However, we did not measure participants’ expectations, which plausibly represented a key mechanism by which negative affectivity affected work centrality. As met expectations have been thought to be central to negative affectivity [43,50], future research addressing the influence of negative affectivity on job attitudes in times of crisis should examine whether (un-)met expectations mediate its effects.

Third, our study did not assess how positive and negative affectivity and work centrality impacted work performance during the pandemic. As there is evidence that fear of COVID-19 negatively affects job performance [68], it would be worth exploring, in the future, how trait affectivity and work centrality jointly influence performance in times of crisis. Fourth, attrition analyses revealed that age reduced the risk of dropping from the sample across time, while being female and positive affectivity increased the risk of leaving the sample at Time 3. These findings are particularly relevant in the case of positive affectivity, as it was a substantive variable in our study. This may have reduced the ability of positive affectivity to predict work centrality. However, no clear post hoc explanation for this finding comes to mind, except that, plausibly, individuals with high positive affectivity may have spent more time with friends and family, hence may have been less available for completing surveys over time. Finally, our study did not account for the possibility that some organizations have implemented supportive practices [10] that may have buffered the negative effects of the fear of COVID-19. Future research may want to consider how organizations adjust their practices during disruptive times, and how this could alter the joint effects of trait affectivity and basic needs satisfaction on job attitudes.

## 5. Conclusions

The present study, partly conducted during the 2020 COVID-19 lockdown in Canada, demonstrates that, contrary to positive affectivity, negative affectivity had unforeseen benefits in terms of increased work centrality over time among employees. Moreover, this positive effect was further amplified when employees experienced a fulfilment of their needs for autonomy and relatedness. We hope the present findings will encourage future endeavors aimed at exploring the unsuspected effects of negative affectivity on work centrality and other outcomes, and how this may be influenced by the satisfaction of various needs.

## Figures and Tables

**Table 1:** Confirmatory Factor Analysis Results: Fit Indices.

	χ^2^(*df*)	NNFI	CFI	RMSEA	SRMR	Δ*χ*^2^(Δ*df*)
1. Hypothesized seven-factor solution	734.67*(278)	0.96	0.97	0.065	0.044	–
2. Six factors: Time 1 positive and negative affectivity combined	1297.30*(284)	0.92	0.93	0.110	0.091	562.63*(6)
3. Six factors: Time 2 and Time 3 work centrality combined	1054.53*(284)	0.94	0.95	0.082	0.053	319.86*(6)
4. Six factors: Time 3 autonomy need satisfaction and Time 3 work centrality combined	1410.21*(284)	0.91	0.92	0.097	0.091	675.54*(6)
5. Six factors: Time 3 relatedness need satisfaction and Time 3 work centrality combined	1402.57*(284)	0.91	0.92	0.097	0.089	667.90*(6)
6. Six factors: Time 3 competence need satisfaction and Time 3 work centrality combined	1437.37*(284)	0.91	0.92	0.098	0.095	702.70*(6)
7. Five factors: Time 3 autonomy, relatedness, and competence need satisfaction combined	2124.03*(289)	0.86	0.88	0.150	0.079	1389.36*(11)
8. One factor: All combined	did not converge					

Note. *N* = 378. NNFI = non-normed fit index; CFI = comparative fit index; IFI = incremental fit index; RMSEA = root-mean-square error of approximation; SRMR = standardized root-mean-square residual. * *p* < 0.001.

**Table 2:** Completely Standardized Confirmatory Factor Analysis Loadings for Scale Items.

Item	Loading
Time 1 Positive affectivity	
1. In general, I feel … inspired	0.70 ***
2. … determined	0.85 ***
3. … active	0.76 ***
4. … attentive	0.75 ***
5. … alert	0.70 ***
Time 1 Negative affectivity	
6. In general, I feel ... upset	0.70 ***
7. ... nervous	0.80 ***
8. ... hostile	0.63 ***
9. ... ashamed	0.57 ***
10. ... afraid	0.76 ***
Time 2 Work centrality	
11. The major satisfaction in my life came from my job	0.91 ***
12. The most important things that happened to me involved my work	0.94 ***
Time 3 Autonomy need satisfaction	
13. The degree of freedom I have to do my job the way I think it can be done best	0.87 ***
14. The opportunities to take personal initiatives in my work	0.91 ***
15. The level of autonomy I have in my job	0.92 ***
16. The opportunities to exercise my own judgement and my own action	0.89 ***
Time 3 Relatedness need satisfaction	
17. The positive social interactions I have at work with other people	0.94 ***
18. The feeling of being part of a group at work	0.95 ***
19. The close friends I have at work	0.94 ***
20. The opportunities to talk with people about things that really matter to me	0.89 ***
Time 3 Competence need satisfaction	
21. The feeling of being competent at doing my job	0.89 ***
22. The level of mastery I can achieve at my task	0.89 ***
23. The level of confidence about my ability to execute my job properly	0.79 ***
24. The sense that I can accomplish the most difficult tasks	0.85 ***
Time 3 Work centrality	
25. The major satisfaction in my life came from my job	0.92 ***
26. The most important things that happened to me involved my work	0.93 ***

Note. *** *p* < 0.001.

**Table 3:** Descriptive Statistics and Correlations among Variables.

Variable	*M*	*SD*	1	2	3	4	5	6	7	8	9	10	11
1. Age	48.10	11.25	–										
2. Gender	1.50	0.50	−0.14 **	–									
3. Education level	2.95	1.43	−0.07	−0.11 **	–								
4. Employment status	1.64	0.48	−0.28 **	−0.06	0.05	–							
5. Positive affectivity (T1)	5.09	0.89	0.21 **	−0.03	0.04	0.00	(0.86)						
6. Negative affectivity (T1)	3.05	0.97	−0.13 **	0.08 *	−0.05	−0.01	−0.26 **	(0.82)					
7. Work centrality (T2)	3.59	1.47	0.06	0.00	0.05	0.10 *	0.12 **	0.08	(0.93)				
8. Autonomy need satis. (T3)	5.23	1.35	0.15 **	−0.10	0.09	0.09	0.28 *	−0.24 **	0.19 **	(0.94)			
9. Relatedness need satis. (T3)	4.80	1.36	0.11 *	−0.12 *	−0.03	0.04	0.24 **	−0.11 *	0.18 **	0.53 **	(0.92)		
10. Compet. need satis. (T3)	5.45	1.29	0.18 **	−0.11 *	−0.00	0.07	0.30 **	−0.26 **	0.13 *	0.72 **	0.58 **	(0.96)	
11. Work centrality (T3)	3.67	1.43	0.13 *	−0.04	0.09	0.02	0.02	0.19 **	0.64 **	0.25 **	0.26 **	0.17 **	(0.92)

Note. *N*s = 379–882. For gender: 1 = male, 2 = female; for education level: 1 = high school, 2 = college, 3 = undergraduate, 4 = certificate, 5 = master, 6 = doctorate in medicine, dentistry, veterinary, or optometry, 7 = Ph.D.; for employment status: 1 = part-time, 2 = full-time; T1 = Time 1; T2 = Time 2; T3 = Time 3. Alpha coefficients are reported on the diagonal in parentheses. * *p* < 0.05; ** *p* < 0.01.

**Table 4:** Results of Moderated Multiple Regression Analyses Predicting Time 3 Work Centrality.

		Model 1		Model 2		Model 3	
Step	Variable(s) Entered	*β*	Δ*R*^2^	*β*	Δ*R*^2^	*β*	Δ*R*^2^
1	Age (T1)	0.06		0.06		0.06	
	Gender (T1)	−0.03		−0.03		−0.03	
	Education level (T1)	0.08 *		0.08 *		0.08 *	
	Employment status (T1)	−0.06		−0.06		−0.06	
	Work centrality (T2)	0.64 ***		0.64 ***		0.64 ***	
			0.43 ***		0.43 ***		0.43 ***
2	Positive affectivity (T1)	−0.01		−0.01		−0.01	
	Negative affectivity (T1)	0.10 *		0.10 *		0.10 *	
			0.01 *		0.01 *		0.01 *
3	Autonomy need satisfaction (T3)	0.17 ***					
			0.02 ***				
	Relatedness need satisfaction (T3)			0.16 ***			
					0.02 ***		
	Competence need satisfaction (T3)					0.13 **	
							0.01 **
4	Positive affectivity × Autonomy need satis.	0.02					
	Negative affectivity × Autonomy need satis.	0.10 *					
			0.01 †				
	Positive affectivity × Relatedness need satis.			0.02			
	Negative affectivity × Relatedness need satis.			0.13 **			
					0.01 **		
	Positive affectivity × Competence need satis.					0.04	
	Negative affectivity × Competence need satis.					0.07	
							0.00

Note. For gender: 1 = male, 2 = female; for education level: 1 = high school, 2 = college, 3 = undergraduate, 4 = certificate, 5 = master, 6 = doctorate in medicine, dentistry, veterinary, or optometry, 7 = Ph.D.; for employment status: 1 = part-time, 2 = full-time; T1 = Time 1; T2 = Time 2; T3 = Time 3. Final model statistics: *F* (10, 377) = 32.40, *p* < 0.001, *R*^2^ = 0.47 (Model 1); *F* (10, 377) = 33.21, *p* < 0.001, *R*^2^ = 0.48 (Model 2); *F* (10, 377) = 30.61, *p* < 0.001, *R*^2^ = 0.46 (Model 3). † *p* < 0.10; * *p* < 0.05; ** *p* < 0.01; *** *p* < 0.001.

## Data Availability

The data presented in this study are available on request from the corresponding author.

## References

[1] Probst T.M., Lee H.J., Bazzoli A. (2020). Economic stressors and the enactment of CDC-recommended COVID-19 prevention behaviors: The impact of state-level context. J. Appl. Psychol..

[2] Bureau of Labor Statistics (2020). Labor Force Statistics from the Current Population Survey. http://data.bls.gov.

[3] Chaney S., King K.U.S. (2020). Jobless claims top 30 millions, as spending, personal income drop. Wall Str. J..

[4] Kumar P., Kumar N., Aggarwal P., Yeap J.A.L. (2021). Working in lockdown: The relationship between COVID-19 induced work stressors, job performance, distress, and life satisfaction. Curr. Psychol..

[5] Trougakos J.P., Chawla N., McCarthy J.M. (2020). Working in a pandemic: Exploring the impact of COVID-19 health anxiety on work, family, and health outcomes. J. Appl. Psychol..

[6] Kniffin K.M., Narayanan J., Anseel F., Antonakis J., Ashford S.P., Bakker A.B., Bamberger P., Bapuji H., Bhave D.P., Choi V.K. (2021). COVID-19 and the workplace: Implications, issues, and insights for future research and action. Am. Psychol..

[7] Prochazka J., Scheel T., Pirozek P., Kratochvil T., Civilotti C., Bollo M., Maran D.A. (2020). Data on work-related consequences of COVID-19 pandemic for employees across Europe. Data Brief.

[8] Becker W.J., Belkin L.Y., Tuskey S.E., Conroy S.A. (2022). Surviving remotely: How job control and loneliness during a forced shift to remote work impacted employee work behaviors and well-being. Hum. Resour. Manag..

[9] Hu J., He W., Zhou K. (2020). The mind, the heart, and the leader in times of crisis: How and when COVID-19-triggered mortality salience relates to state anxiety, job engagement, and prosocial behavior. J. Appl. Psychol..

[10] Mihalache M., Mihalache O.R. (2022). How workplace support for the COVID-19 pandemic and personality traits affect changes in employees’ affective commitment to the organization and job-related well-being. Hum. Resour. Manag..

[11] Judge T.A., Erez A., Bono J.E., Thoresen C.J. (2003). The Core Self-Evaluations Scale: Development of a measure. Pers. Psychol..

[12] Wilmot M.P., Wanberg C.R., Kammeyer-Mueller J.D., Ones D.S. (2019). Extraversion advantages at work: A quantitative review and synthesis of the meta-analytic evidence. J. Appl. Psychol..

[13] Hao L., Meng W., Xu M., Meng H. (2022). Work centrality and recovery experiences in dual-earner couples: Test of an actor-partner interdependence model. Stress Health.

[14] Hirschfeld R.R., Feild H.S. (2000). Work centrality and work alienation: Distinct aspects of a general commitment to work. J. Organ. Behav..

[15] Diefendorff J.M., Brown D.J., Kamin A.M., Lord R.G. (2002). Examining the roles of job involvement and work centrality in predicting organizational citizenship behaviors and job performance. J. Organ. Behav..

[16] Jiang L., Johnson M.J. (2018). Meaningful work and affective commitment: A moderated mediation model of positive work reflection and work centrality. J. Bus. Psychol..

[17] Klussman K., Nichols A.L., Langer J. (2021). Mental health in the United States during the COVID-19 pandemic: A longitudinal examination of the ameliorating effect of meaning salience. Curr. Psychol..

[18] Fredrickson B.L. (2001). The role of positive emotions in positive psychology: The broaden-and-build theory of positive emotions. Am. Psychol..

[19] Watson D., Wiese D., Vaidya J., Tellegen A.E. (1999). The two general activation systems of affect: Structural findings, evolutionary considerations, and psychobiological evidence. J. Personal. Soc. Psychol..

[20] Deci E.L., Olafsen A.H., Ryan R.M. (2017). Self-determination theory in work organizations: The state of a science. Annu. Rev. Organ. Psychol. Organ. Behav..

[21] Ryan R.M., Deci E.L. (2000). Self-determination theory and the facilitation of intrinsic motivation, social development, and well-being. Am. Psychol..

[22] Van den Broeck A., Ferris D.L., Chang C.H., Rosen C.C. (2016). A review of self-determination theory’s basic psychological needs at work. J. Manag..

[23] Vansteenkiste M., Ryan R.M., Soenens B. (2020). Basic psychological need theory: Advancements, critical themes, and future directions. Motiv. Emot..

[24] Shao R., He L., Chang C., Wang M., Baker N., Pan J., Jin Y. (2021). Employees’ reactions towards COVID-19 information exposure: Insights from terror management theory and generativity theory. J. Appl. Psychol..

[25] Ng T.W.H., Sorensen K.L. (2009). Dispositional affectivity and work-related outcomes: A meta-analysis. J. Appl. Soc. Psychol..

[26] Thoresen C.J., Kaplan S.A., Barsky A.P., Warren C.R., de Chermont K. (2003). The affective underpinnings of job perceptions and attitudes: A meta-analytic review and integration. Psychol. Bull..

[27] Radanielina-Hita M.-L., Grégoire Y., Lussier B., Boissonneault S., Vandenberghe C., Sénécal S. (2023). An extended health belief model for COVID-19: Understanding the media-based processes leading to social distancing and panic buying. J. Acad. Mark. Sci..

[28] Watson D., Clark L.A., Tellegen A. (1988). Development and validation of brief measures of positive and negative affect: The PANAS Scales. J. Personal. Soc. Psychol..

[29] Barsky A., Thoresen C.J., Warren C.R., Kaplan S.A. (2004). Modeling negative affectivity and job stress: A contingency-based approach. J. Organ. Behav..

[30] Bowling N.A., Hendricks E.A., Wagner S.H. (2008). Positive and negative affectivity and facet satisfaction: A meta-analysis. J. Bus. Psychol..

[31] Carver C.S. (2006). Approach, avoidance, and the self-regulation of affect and action. Motiv. Emot..

[32] Carver C.S., White T.L. (1994). Behavioral inhibition, behavioral activation, and affective responses to impending reward and punishment: The BIS/BAS Scales. J. Personal. Soc. Psychol..

[33] Gray J.A. (1990). Brain systems that mediate both emotion and cognition. Cogn. Emot..

[34] Fredrickson B.L. (2009). Positivity.

[35] Fredrickson B.L., Cohn M.A., Coffey K.A., Pek J., Finkel S.M. (2008). Open hearts build lives: Positive emotions, induced through loving-kindness meditation, build consequential personal resources. J. Personal. Soc. Psychol..

[36] Vandenberghe C., Panaccio A., Bentein K., Mignonac K., Roussel P., Ben Ayed A.K. (2019). Time-based differences in the effects of positive and negative affectivity on perceived supervisor support and organizational commitment among newcomers. J. Organ. Behav..

[37] Schweitzer V.M., Rivkin W., Gerpott F.H., Diestel S., Kühnel J., Prem R., Wang M. (2022). Some positivity per day can protect you a long way: A within-person field experiment to test an affect-resource model of employee effectiveness at work. Work Stress.

[38] Fredrickson B.L., Devine P., Plant A. (2013). Positive emotions broaden and build. Advances in Experimental Social Psychology.

[39] Schutte N.S. (2014). The broaden and build process: Positive affect, ratio of positive to negative affect and general self-efficacy. J. Posit. Psychol..

[40] Fredrickson B.L., Branigan C. (2005). Positive emotions broaden the scope of attention and thought-action repertoires. Cogn. Emot..

[41] Schaubroeck J., Ganster D.C., Fox M.L. (1992). Dispositional affect and work-related stress. J. Appl. Psychol..

[42] Lonigan C.J., Vasey M.W. (2009). Negative affectivity, effortful control, and attention to threat-relevant stimuli. J. Abnorm. Child Psychol..

[43] Begley T., Lee C. (2005). The role of negative affectivity in pay-at-risk reactions: A longitudinal study. J. Appl. Psychol..

[44] Vandenberghe C., Panaccio A., Ben Ayed A.-K. (2011). Continuance commitment and turnover: Examining the moderating role of negative affectivity and risk aversion. J. Occup. Organ. Psychol..

[45] Wanous J.P., Poland T.D., Premack S.L., Davis K.S. (1992). The effects of met expectations on newcomer attitudes and behaviors: A review and meta-analysis. J. Appl. Psychol..

[46] Ryan R.M., Deci E.L. (2017). Self-Determination Theory: Basic Psychological Needs in Motivation, Development, and Wellness.

[47] Van den Broeck A., Vansteenkiste M., De Witte H., Soenens B., Lens W. (2010). Capturing autonomy, competence, and relatedness at work: Construction and initial validation of the Work-Related Basic Need Satisfaction Scale. J. Occup. Organ. Psychol..

[48] Baumeister R.F., Leary M.R. (1995). The need to belong: Desire for interpersonal attachments as a fundamental human motivation. Psychol. Bull..

[49] Kuvaas B., Buch R., Weibel A., Dysvik A., Nerstad C.G. (2017). Do intrinsic and extrinsic motivation relate differently to employee outcomes?. J. Econ. Psychol..

[50] Panaccio A., Vandenberghe C., Ben Ayed A.K. (2014). The role of negative affectivity in the relationships between pay satisfaction, affective and continuance commitment, and voluntary turnover: A moderated mediation model. Hum. Relat..

[51] Cheung J.H., Burns D.K., Sinclair R.R., Sliter M. (2017). Amazon Mechanical Turk in organizational psychology: An evaluation and practical recommendations. J. Bus. Psychol..

[52] DeSimone J.A., Harms P.D. (2018). Dirty data: The effects of screening respondents who provide low-quality data in survey research. J. Bus. Psychol..

[53] Goodman J.S., Blum T.C. (1996). Assessing the non-random sampling effects of subject attrition in longitudinal research. J. Manag..

[54] Thompson E.R. (2007). Development and validation of an internationally reliable short-form of the positive and negative affect schedule (PANAS). J. Cross-Cult. Psychol..

[55] Chiniara M., Bentein K. (2016). Linking servant leadership to individual performance: Differentiating the mediating role of autonomy, competence and relatedness need satisfaction. Leadersh. Q..

[56] Bal P.M., Kooij D. (2011). The relations between work centrality, psychological contracts, and job attitudes: The influence of age. Eur. J. Work Organ. Psychol..

[57] Li J., Huang M.-T., Hedayati-Mehdiabadi A., Wang Y., Yang X. (2020). Development and validation of work ethic instrument to measure Chinese people’s work-related values and attitudes. Hum. Resour. Dev. Q..

[58] Jöreskog K.G., Sörbom D., Du Toit S., Du Toit M. (2001). LISREL 8: New Statistical Features.

[59] Hu L.T., Bentler P.M. (1999). Cutoff criteria for fit indexes in covariance structure analysis: Conventional criteria versus new alternatives. Struct. Equ. Model..

[60] Kline R.B. (2015). Principles and Practice of Structural Equation Modeling.

[61] Aguinis H., Gottfredson R.K. (2010). Best-practice recommendations for estimating interaction effects using moderated multiple regression. J. Organ. Behav..

[62] Cohn M.A., Fredrickson B.L., Brown S.L., Mikels J.A., Conway A.M. (2009). Happiness unpacked: Positive emotions increase life satisfaction by building resilience. Emotion.

[63] Fredrickson B.L., Losada M.F. (2005). Positive affect and the complex dynamics of human flourishing. Am. Psychol..

[64] Prinzing M.M., Zhou J., West T.N., Le Nguyen K.D., Wells J.L., Fredrickson B.L. (2022). Staying ‘in sync’ with others during COVID-19: Perceived positivity resonance mediates cross-sectional and longitudinal links between trait resilience and mental health. J. Posit. Psychol..

[65] Tang W.-G., Vandenberghe C. (2020). Is affective commitment always good? A look at within-person effects on needs satisfaction and emotional exhaustion. J. Vocat. Behav..

[66] Stanley P.J., Schutte N.S. (2023). Merging the Self-Determination Theory and the Broaden and Build Theory through the nexus of positive affect: A macro theory of positive functioning. New Ideas Psychol..

[67] Waters L., Algoe S.B., Dutton J., Emmons R., Fredrickson B.L., Heaphy E., Moskowitz J.T., Neff K., Niemiec R., Pury C. (2022). Positive psychology in a pandemic: Buffering, bolstering, and building mental health. J. Posit. Psychol..

[68] Raja U., Jahanzeb S., Malik M.A.R., Baig M.U.A. (2022). Dispositional causes of burnout, satisfaction, and performance through the fear of COVID-19 during times of pandemic. Appl. Psychol. Int. Rev..

